# Differential diffusion of pharmaceutical innovations in a mixed market middle - income economy

**DOI:** 10.1186/s12913-021-06786-6

**Published:** 2021-10-19

**Authors:** Nurhafiza Md Hamzah, Kok Fong See

**Affiliations:** 1 Planning Division, Ministry of Health Malaysia, Federal Administrative Centre, Level 3, Block E6, E Complex, 62590 Putrajaya, Malaysia; 2 Economics Programme, School of Distance Education, Universiti Sains Malaysia, 11800 Minden, Pulau Pinang Malaysia

**Keywords:** Panel regression, Medicines utilization, Diffusion, New Chemical Entities, Adoption

## Abstract

**Background:**

Policymakers are faced with the challenge of balancing patient’s access for effective and affordable medicines to sustain the rising healthcare costs. In a mixed healthcare market such as Malaysia, coverage decisions of new medicines are different: public funded health system has a formulary listing process whereas for private sector, which is a market-based economy, depends on patient’s willingness to pay and insurance coverage. There is little overlap between public and private healthcare service delivery with access to new innovative medicines, as differentiated by sources of funding. The objectives of this study were to examine the diffusion of New Chemical Entities (NCEs) into the public and private healthcare market between 2010 and 2014, and determine the factors explaining the diffusion.

**Methods:**

We matched medicines from the product registration database by medicine formulation to medicines in IQVIA National Pharmaceutical Audit database for each year. The price per Defined Daily Dose (DDD), market concentration and generic utilization share variables were calculated. A panel fixed effect model was performed to measure diffusion of NCEs for each year and test possible determinants of diffusion of NCEs for overall market and sector specifics.

**Results:**

The utilization of NCEs was larger in the private sector compared to the public sector but the speed of diffusion over time was higher in the public sector. Price per DDD was negatively associated with diffusion of NCEs, while generic utilization share was significantly regressive in the public sector. Market concentration was negatively associated with utilization of NCEs, however result tends to be mixed according to sector and Anatomical Therapeutic Chemical (ATC) category.

**Conclusions:**

Understanding key aspects of sectoral variation in diffusion of NCEs are crucial to reduce the differences of access to new medicines within a country and ensure resources are used on cost effective treatments.

**Supplementary Information:**

The online version contains supplementary material available at 10.1186/s12913-021-06786-6.

## Background

Malaysia has mixed government - funded public health service delivery with parallel private health service delivery funded from private funds [1]. Public healthcare services are financed through general taxation (2.2 % of gross domestic product [GDP] in 2018) while private healthcare services are funded by household out of pocket payments (72 % of total expenditure on private healthcare), private insurance and employers [2]. Public healthcare system is vital as utilization of healthcare services is larger, with public hospitals treating 86 % of total outpatient attendance and 77 % of total inpatient admissions [3]. Pharmaceuticals represent a sizable share of healthcare expenditure in Malaysia, with 12 % of total health spending in 2017 [2, 4]. Similar to healthcare utilization trends, public sector medicines supply accounts for 67 % of prescription medicines volume in Malaysia, yet only spends 33 % of total share of prescription medicines expenditure [5].

Rapid development of drug treatment in few therapeutic areas are observed in recent years due to pharmaceutical innovation such as targeted therapies and biopharmaceuticals. The introduction of these new therapies improves population health but is also expensive and contributed to the growth in pharmaceutical spending, thus putting increasing pressure on the health system. Generally, pharmaceutical manufacturers will apply for regulatory approval to the national regulatory authority of countries that they plan to market their medicines. On average, nearly 60 new chemical entities (NCEs) are registered in the Malaysian pharmaceutical market annually [6]. Diffusion of NCE is important for patients to have access to new innovative medicines that potentially be more effective in treating their disease. In a mixed healthcare market such as Malaysia, coverage decisions of new medicines are different: public funded health system has a formulary listing process for medicines to be distributed within the system whereas for private sector, a market-based economy, depends on patient’s willingness to pay and insurance coverage. There is little overlap between public and private healthcare service delivery with access to new innovative medicines, as differentiated by sources of funding.

The concept of diffusion of an innovation relies on the ‘Diffusion of Innovations Theory’ as a process which an innovation is communicated through channels for a period of time among the members of a social system [7]. The theory suggests an innovation that is viewed as advantageous by its early adopters will increase steadily over time and form an adoption curve that is S - shaped. [8]. In fact, Bonair and Persson framework [9] proposed that the determinants of diffusion can be categorized into three main areas: 1) characteristics of the innovation, 2) actors in the process and 3) structure and environment [10]. The characteristics of an innovation are derived if the new medicines are perceived as having greater relative advantage, compatibility, trialability, observability and less complexity that would result in it being adopted more rapidly than other medicines [10]. The actors in the process are healthcare providers, including physicians and health care organizations, along with decision makers, such as policy makers and regulators, public and private insurers, patients and manufacturers [10]. Finally, the structure and environment covers the organizational characteristics of potential adopters, financing of health services and legal regulations or guidelines surrounding the adoption of health technologies [11]. The complexity of the process is highlighted by Atun et al. [12] in which collective interaction of the health systems characteristics, individual health institutions and adopting entities influences the receptiveness of health systems to adopt new innovation, as well as the speed and scale of the diffusion [12, 13].

To date, there have been a comprehensive analysis on the determinants of diffusion of medicines. Most studies analysed from two levels of aggregation: at the market / industry level or from the individual decision - maker perspective. At the market / industry level, factors such as regulatory policies [14–24] and market characteristics [25] have been studied whereas at individual level, prescriber’s characteristics [26–28] and organisational - related factors [29] were assessed. Most studies investigated the diffusion of medicines within a specific country [30–32] but some studies have made international comparisons [16, 25, 33]. The following discussion provides a brief review of empirical studies assessing factors on regulatory and market characteristics associated with diffusion of medicines. Desiraju et al. (2004) [25] studied the diffusion of new pharmaceuticals in 15 developed and developing countries found that developing countries had lower diffusion speed compared to developed countries. Their findings also showed that per - capita expenditures on health care had a positive effect on diffusion speed (particularly for developed countries), while higher prices led to decrease in diffusion speed. Similar study by Brekke et al. [33] assessed the diffusion of anti - TNF drugs over nine European countries from the year of first launch in 2000 until 2009 showed that the cross-country variations can be explained by the time - invariant country specific factors (e.g., disease prevalence, demographics, health care system). They also observed consistent findings that the cross - country variations in consumption were attributed by the differences in income (GDP per capita) and health spending (share of GDP). Berndt et al. (2007) [16] explored the diffusion of new drugs across 15 countries in three therapeutic classes (antihypertensives, antidepressants, antiepileptics) between 1992 and 2003 found substantial heterogeneity across therapeutic classes and countries in diffusion of new medicines. In a different study by Costa-Font, et al. [34], 13 different therapeutic categories were assessed between 1999 and 2008 in 20 Organisation for Economic Co-operation and Development countries showed that price and reimbursement regulations delayed the adoption of new pharmaceutical products. A comprehensive review on the available empirical evidence on the diffusion of new medicines discovered that the evidence is limited to only two of the most related factors in the diffusion of pharmaceutical innovations; intellectual property rights and pharmaceutical market characteristics [35]. This is supported by Atun and Gurol - Urganci [15] that acknowledged the shortage of literature on regulatory policies and health system interactions that influence the adoption and diffusion of innovative medicines.

The paper differs from almost all previous studies in several important ways. Although previous empirical studies have assessed cross - national differences in the diffusion of new medicines, relatively little published evidence were found concerning variations in the utilization, and differential rates of diffusion of new medicines in a mixed healthcare market within a country. This study fills a gap in the literature by exploring public - private sectoral differences in the diffusion of pharmaceutical innovations in a mixed market upper middle - income economy. The objectives of this study are to examine the diffusion of NCEs in the public and private healthcare market and determine the factors explaining the diffusion. The outline of the paper is as follows: Methods section briefly explains the methodology and the model. In Results section we present the data and the estimation results. Finally, Discussion section concludes the paper.

## Methods

**Fig. 1:**
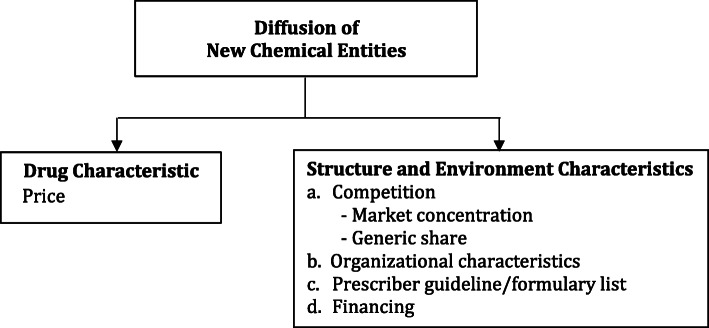
Conceptual framework of assessing diffusion of NCEs

We adapted the Bonair and Persson framework to assess the diffusion of NCEs between public and private sector in Malaysia as described in Fig. 1. The price of NCEs is used to capture the difference between public and private sector in terms of drug characteristic. We assessed the structure and environment characteristics using market concentration and generic share utilization variables in both public and private sectors. In addition, other sector specific information related to the analysis is further explained in Table 1.

**Table 1:** Structure and environment characteristics between public and private sector

	Public	Private
Organizational Characteristics	a. Centralized procurement procedure which includes: - National tenders - Price negotiations b. Generic medicines policy	a. Fragmented procurement b. Generic medicines policy is not mandatory.
Prescriber / formulary list	a. Standard list b. Centralize coverage decision for cost effective intervention	a. List differ by individual facilities b. Coverage decision by individual facilities
Financing	a. Fixed health budget b. Tax based c. User fee is highly subsidized	a. Out of pocket b. Private health insurance c. Fee-for-service payment

We explore the factors associated with the diffusion of an NCE within a mixed public – private pharmaceutical market over 5 years from 2010 to 2014. As an innovative product, NCE is considered to have diffused based on its (increasing) utilization over time. Since our dataset contains product – level panel dataset consists of detailed sales information of NCE products in public and private sector over 5 years, we can control for time invariant product – and market – specific factors (both observed and unobserved). Generally, static panel data regression model includes pooled Ordinary Least Square (OLS), fixed effects (FE) model and random effects (RE) model. In order to identify the most appropriate model for the study, three different tests, i.e., Hausman test, Chow Test and Breusch and Pagan Lagrangian multiplier test were adopted in the study (Annex, Table 1). From these results, we concluded that different intercept exists among products and hence FE model is more appropriate for the estimation. Information on data source, variable construction and model specification is described in the next section.

### Data sources

#### Quest 3 + Product Registration Database

We obtained product registration data from 2009 to 2014 from the Quest 3 + system, an integrated online product registration system under the National Pharmaceutical Regulatory Agency (NPRA) Malaysia. This Quest 3 + dataset of NCE products contains the year of registration approval, active ingredients, product name, dosage form and strength.

#### IQVIA Malaysia Pharmaceutical Audit

Data on products sales for 2010 to 2014 was acquired from IQVIA (formerly known as QuintilesIMS) that collects data from main pharmaceutical distributors in Malaysia. This dataset includes information such as sales volumes and values for each product pack, with other relevant information such as active ingredients, strength, dosage form and pack sizes.

#### Variable construction

NCE is defined by the NPRA either as 1) active moiety / radiopharmaceutical substance that has not been registered in any pharmaceutical product or 2) Hybrid NCE that is single / combination products with registered active moieties [36]. NCEs registered in the Quest 3 + dataset were matched to medicinal products from the IQVIA Pharmaceutical Audit data by medicine formulation (active ingredient, dosage form and strength). After matching of products was performed, explanatory variables for each year were constructed as follows:

**Utilization**, the outcome variable was calculated as the natural log of Defined Daily Doses (DDDs)^2^ per 1,000 population for the year. DDDs per 1000 capita per year were not normally distributed and hence log transformation was used to improve homoskedasticity.

**Price per DDD** was calculated as the annual average ex manufacturer/wholesale weighted (by volume) price (in MYR^3^) for all the NCE products deflated with DDDs. The price per DDD was computed to reflect the average daily dose price for a typical patient, noting that the product, form and strength may vary according to the usage over time. For the purpose of statistical analyses, the natural log of price per DDD was used in the model.

**Generic utilization share.** A generic product contains the same chemical entity as an existing innovator and hence acts as a substitute for the innovator and medicinal products within the same therapeutic class. We calculated generic utilization (volume deflated by DDD) share as the proportion of generics used within the same therapeutic group (ATC level 4).

**Market concentration – Herfindahl – Hirschman Index (HHI).** We measured pharmaceutical market concentration using HHI within the same therapeutic group. *HHI* is defined as $HHI={\sum }_{i=1}^{N}\left({{m}_{i}}^{2}\right)$, where *m* is the market share of NCE *I* and *N* is the number of NCE products in the same therapeutic group [37]. The ATC4^4^ classification is used to define the potential market group as the effect of competition and substitution are assumed strongest at this level [34]. A high HHI indicates that the market is concentrated among few large NCEs and hence new medicines would face high barriers of entry.

**Year(s) since product registration** is used to capture the time dimension for diffusion. The longer the time duration, the higher the medicines will diffuse. Conversely, as time passes, new competitors that enter the market to compete against the molecule are higher, lowering its comparative therapeutic advantage [37].

### Model specification

Three FE models were specified for the analyses. Model 1 pools together NCE utilization from both the public and private sector panels to explore factors for overall market, and is specified as:

$$\text{ln}{Y}_{it}={\alpha }_{i}+ {\beta }_{1}ln{P}_{it}+ {\beta }_{2}{HHI}_{it}+ {\beta }_{3}{GS}_{it} +\sum _{j= 1}^{5}{{\gamma }_{j}YR}_{ij}{+u}_{it}$$

where Y_it_ is NCE product i utilization per 1,000 capita in year t, P_it_ is the average NCE product i price per DDD in year t, HHI_it_ is the market concentration at ATC– 4 of NCE product i in year t, GS_it_ is the generic utilization share by DDD at ATC– 4 of product i in year t, YR_ij_ is the number of years since registration for NCE product i and u_it_ is the error term consists of the individual specific random component (µi) and idiosyncratic disturbance (ε_it_).

Models 2(a) and 2(b) explore the differential effects on diffusion of broad ATC categories separately for the public sector (model 2a) and private sector (model 2b)$.$

Model 2(a) Public sector

$$\text{ln}{Y}_{{public}_{it}}={\alpha }_{i}+ {\beta }_{1}ln{P}_{{public}_{it}}+ {\beta }_{2}{HHI}_{{public}_{it}}+ {\beta }_{3}{GS}_{{public}_{it}}+\sum _{j= 1}^{5}{{\gamma }_{j}YR}_{{public}_{ij}}{+u}_{it}$$

Model 2(b) Private sector

$$\text{ln}{Y}_{{private}_{it}}={\alpha }_{i}+ {\beta }_{1}ln{P}_{{private}_{it}}+ {\beta }_{2}{HHI}_{{private}_{it}}+ {\beta }_{3}{GS}_{{private}_{it}}+\sum _{j= 1}^{5}{{\gamma }_{j}YR}_{{private}_{ij}}{+u}_{it}$$

where variables are defined in model 1 above.

Model 3 and 4 explore two ATC level 1 categories separately – C – Cardiovascular system and L – Antineoplastic agents and immunomodulating agents – to tease out sectoral differences for two key categories of pharmaceutical agents to treat cardiovascular diseases and cancers. Generally, cardiovascular diseases such as hypertension and hypercholesterolemia affect a much larger proportion of the population and require lifelong treatment compared to cancers, which affect a smaller proportion of the population and treatment regime such as chemotherapy are episodic. Similar to previous models, panel regression for cardiovascular system (ATC C) and antineoplastic agents and immunomodulating agents (ATC L) can be estimated for all sectors and sector-specific using abovementioned equations. Statistical analyses were conducted using Stata 15 [38].

#### Limitations

Some NCEs are relevant for treating a large proportion of the population while some have much narrower indications and are used by much fewer numbers. Model 3 and 4 serve respectively as proxies to tease out differential findings for these two polarities.

#### Robustness checks

Several diagnostics including variance inflation factor test (for multicollinearity), Wooldridge serial correlation test (for autocorrelation) and Breusch– Pagan Lagrangian Multiplier test (for heteroskedasticity) were carried out to ensure model assumptions were met. Considering that the issue of autocorrelation and heteroskedasticity do exists, robust standard errors are more appropriate to be used in the analyses. The regression estimated robust standard errors to take into account cluster groups as a result of medicinal products (e.g., same brand) that occur across all five years of the study.

## Results

From the period under study (2010 to 2014), 210 (57 %) NCE formulations were adopted out of 367 registered – of which 180 (49 %) and 193 (53 %) formulations were diffused in the public sector and private sector, respectively. The formulations adopted that are common to both sectors were 171 and this subset of NCE formulations was included in the analysis.

### Descriptive statistics

Table 2 reports the descriptive statistics. By examining the number of NCE unique formulations, the highest number of formulations within a therapeutic group available in both sectors was the nervous system (ATC N) followed by antineoplastics and immunomodulating agents (ATC L). By value, the bulk of the NCEs in the public sector were nervous system (ATC N), blood and blood forming agents (ATC B) and alimentary tract and metabolism (ATC A) whereas in the private sector, the highest expenditure categories were alimentary tract and metabolism (ATC A) medicines, cardiovascular (ATC C) medicines and blood and blood forming agents (ATC B). For matched formulations in both public and private sectors, the highest expenditure categories were alimentary tract and metabolism (ATC A), nervous system (ATC N) and cardiovascular (ATC C) medicines. In terms of utilization, the largest volume of NCEs used in the public sector were alimentary and metabolism (ATC A), genitourinary system and sex hormones (ATC G), and nervous system (ATC N) medicines. For private sector and matched formulations, the highest utilization of NCEs were found in alimentary tract and metabolism (ATC A), cardiovascular (ATC C), and genitourinary system and sex hormones (ATC G) medicines. In summary, we observe slight differences in expenditure and utilization of NCEs in the public and private sector.

**Table 2:** NCE formulations

ATC Level 1 Category	Number of NCEs (% of total medicines)			Expenditure (% of total values)			Utilization (% of total DDDs)		
	Public *n* = 180	Private *n* = 193	Panel *n* = 171	Public *n *= 180	Private *n* = 193	Panel *n* = 171	Public *n* = 180	Private *n* = 193	Panel *n* = 171
A – Alimentary tract and metabolism	13.9	14.0	12.9	16.8	34.9	27.3	67.5	49.5	52.1
B – Blood and blood forming organs	11.7	11.9	11.7	17.8	12.1	14.5	2.8	4.0	4.0
C – Cardiovascular system	11.7	10.4	11.7	4.5	21.1	15.5	7.4	25.5	20.9
D – Dermatalogicals	1.1	1.0	1.2	0.3	0.8	0.6	0.2	0.8	0.6
G – Genito – urinary system and sex hormones	7.2	9.3	7.6	6.4	11.1	9.1	12.6	12.9	13.9
J – Anti-infectives for systemic use	5.0	3.6	4.1	2.0	1.9	2.0	0.7	0.4	0.5
L – Antineoplastic agents and immunomodulating agents	17.8	18.7	18.7	10.0	8.8	9.4	0.3	1.0	0.8
M – Musculo – skeletal system	2.2	2.1	2.3	0.4	1.5	1.2	0.4	1.4	1.1
N – Nervous system	22.8	20.7	23.4	41.4	5.3	18.7	7.9	1.6	4.3
R – Respiratory system	5.6	5.7	5.8	0.5	2.3	1.7	0.4	2.4	1.8
S – Sensory Organs	0.6	1.6	0.6	< 0.1	0.3	< 0.1	< 0.1	0.5	< 0.1

Table 3 summarizes the key variables included in the analysis. The private sector had more utilization of NCEs registered in 2009 to 2014 by an average increase of 51 % compared to the public sector. In fact, the private sector had a higher average price per DDD per 1000 capita compared to public sector. On the other hand, the public sector market was more concentrated (HHI of 0.37 vs. 0.32) and had a higher generic utilization share (389 vs. 277 total DDDs) than the private sector.

**Table 3:** Summary statistics

Variable		Parameters			
		Public (*n* = 531)		Private (*n* = 615)	
Utilization – DDD per 1,000 capita per year, Mean (SD)		5.04 (34.38)		10.18 (47.46)	
Price per DDD per 1,000 capita, Mean (SD)		2.43 (14.20)		2.68 (15.73)	
Market Concentration (HHI) at ATC-4, Mean (SD)		0.37 (0.23)		0.32 (0.23)	
Generic Competition (Total DDDs), Mean (SD)		389.15 (1815.12)		276.66 (979.18)	
**Therapeutic group at ATC Level 1, N (%)**					
A – Alimentary tract and metabolism		57 (10.73)		68 (11.06)	
B – Blood and blood forming organs		69 (12.99)		75 (12.20)	
C – Cardiovascular system		65 (12.24)		77 (12.52)	
D – Dermatalogicals		8 (1.51)		10 (1.63)	
G – Genito – urinary system and sex hormones		43 (8.10)		50 (8.13)	
J – Antiinfectives for systemic use		22 (4.14)		22 (3.58)	
L – Antineoplastic agents and immunomodulating agents		93 (17.51)		109 (17.72)	
M – Musculo – skeletal system		8 (1.51)		14 (2.28)	
N – Nervous system		142 (26.74)		158 (25.69)	
R – Respiratory system		23 (4.33)		30 (4.88)	
S – Sensory Organs		1 (0.19)		2 (0.33)	
**Year(s) since registration, N (%)**					
Year 1		118 (22.22)		149 (24.23)	
Year 2		136 (25.61)		149 (24.23)	
Year 3		116 (21.85)		118 (19.19)	
Year 4		96 (18.08)		98 (15.93)	
Year 5		43 (8.10)		48 (7.80)	

Note: The values are mean and standard deviation (in parentheses)

### Panel Regression Estimates

#### All - sector Regression Estimates

Table 4 for Model 1 reports all – sector regression estimates. The results indicated that the estimated price elasticity of demand is elastic and significant. The generic share coefficient was significant and negatively related to utilization. Market concentration had a significantly negative effect on utilization which indicates that the higher the competition in the pharmaceutical market, the higher the diffusion of NCEs. To further illustrate, the estimated marginal effect indicates that the utilization of NCEs will increase by 0.4 percentage points when an additional product enters the same therapeutic market if all factors held constant.

**Table 4:** Factors that affects the diffusion of NCE products: Panel regression results for all therapeutic groups

Model 1				Model 2(a) and Model 2(b)						
					Public			Private		
**Variable**	**Coefficient**		**Standard Error**	**Variable**	**Coefficient**		**Standard Error**	**Coefficient**		**Standard Error**
Intercept	0.964		(1.433)	Intercept	1.926		(1.456)	-1.499	***	(2.426)
**Drug characteristic**										
ln price per DDD	-1.326	**	(0.489)	ln price per DDD	-1.874	***	(0.473)	-0.291	***	(0.858)
**Structure and environment characteristics**										
Generic share (DDD)	-1.237	*	(0.490)	Generic share (DDD)	-2.390	***	(0.583)	0.131		(0.625)
Market concentration (HHI)	-1.383	*	(0.553)	Market concentration (HHI)	-1.305		(0.723)	-1.773	*	(0.870)
**Year after Registration**										
Year 1	1.467	***	(0.189)	Year 1	1.222	***	(0.295)	1.596	***	(0.187)
Year 2	2.347	***	(0.205)	Year 2	2.299	***	(0.309)	2.313	***	(0.198)
Year 3	2.685	***	(0.216)	Year 3	2.843	***	(0.324)	2.440	***	(0.213)
Year 4	2.944	***	(0.232)	Year 4	3.194	***	(0.338)	2.604	***	(0.221)
Year 5	3.126	***	(0.234)	Year 5	3.313	***	(0.343)	2.915	***	(0.233)
*R* - squared	0.610			*R* - squared	0.529			0.344		
Corr (u_i, Xb)	-0.424			Corr (u_i, Xb)	-0.802			0.305		
Rho	0.863			Rho	0.938			0.917		
Number of observations	1,146			Number of observations	531			615		

* *P*<0.05; ** *P*<0.01; *** *P*<0.001

### Sector - specific Regression Estimates

Model 2 reports separate regressions by sector which permits estimated coefficients to be sector specific. The sector-specific regressions confirmed that price is elastic in public sector and inelastic in private sector with significant estimates. Generic share was significantly related to utilization in the public sector but not significant in the private sector. In contrast, market concentration was significantly associated with the private sector, but effect was not significant in the public sector. The generic use in the private sector indicates lack of generic competition within therapeutic groups. As for public sector, each additional generic competitor as a substitute will reduce NCE utilization by 1.1 percentage points if all variables remain constant. The separate regression results demonstrated that the public sector coefficients strongly influence the overall regression effects. The separate regression also confirms that changes in the rate of diffusion over the years was larger in the public sector while changes in the rate of diffusion was smaller in the private sector. The utilization increased by 2.7 percentage points over time from the base year in public sector whereas utilization increment of 1.8 percentage points over time from the base year was observed in the private sector.

### ***Comprehensive anti - neoplastics*** and cardiovascular class results

We selected two categories; Antineoplastic and immunomodulating medicines, and cardiovascular medicines for more detailed analysis as these groups represent the highest number of NCEs in the market and reflect the current non communicable disease burden in Malaysia. Tables 5 and 6 report panel regression results for anti - neoplastics and immunomodulating medicines (ATC L class) and cardiovascular medicines (ATC C class).

**Table 5:** Factors that affects the diffusion of NCE products: Panel regression for Antineoplastic agents and immunomodulating agents (ATC L)

Model 3				Model 3(a) and Model 3(b)						
					**Public**			**Private**		
Variable	**Coefficient**		**Standard Error**	**Variable**	**Coefficient**		**Standard Error**	**Coefficient**		**Standard Error**
Intercept	1.892		(2.124)	Intercept	4.299		(3.509)	-0.768		(2.189)
**Drug characteristic**										
Ln price per DDD	-1.186	**	(0.379)	Ln price per DDD	-1.588	*	(0.661)	-0.821		(0.405)
**Structure and environment characteristics**										
Generic share (DDD)	-2.222	*	(1.062)	Generic share (DDD)	-4.279	***	(0.623)	-0.256		(0.492)
Market concentration (HHI)	-3.094	*	(1.314)	Market concentration (HHI)	-4.194	**	(1.441)	-2.263		(1.134)
**Year after registration**										
Year 1	1.566	**	(0.495)	Year 1	1.341	***	(0.267)	1.826	***	(0.511)
Year 2	2.530	***	(0.460)	Year 2	2.504	***	(0.187)	2.568	***	(0.491)
Year 3	2.696	***	(0.519)	Year 3	2.786	***	(0.278)	2.636	***	(0.608)
Year 4	2.959	***	(0.563)	Year 4	3.023	***	(0.434)	3.008	***	(0.587)
Year 5	3.199	***	(0.581)	Year 5	3.015	***	(0.382)	3.540	***	(0.601)
R – squared	0.788			R - squared	0.766			0.780		
Corr (u_i, Xb)	-0.535			Corr (u_i, Xb)	-0.855			0.284		
Rho	0.841			Rho	0.932			0.859		
Number of observations	202			Number of observations	93			109		

* *P* < 0.05; ** *P* < 0.01; *** *P* < 0.001

**Table 6:** Factors that affects the diffusion of NCE products: Panel regression for the cardiovascular system (ATC C)

Model 4				Model 4(a) and Model 4(b)						
					**Public**			**Private**		
**Variable**	**Coefficient**		**Standard Error**	**Variable**	**Coefficient**		**Standard Error**	**Coefficient**		**Standard Error**
Intercept	0.587		(0.848)	Intercept	-4.564	*	(2.015)	-0.303		(1.643)
**Drug characteristic**										
Ln price per DDD	-2.782	***	(0.703)	Ln price per DDD	-1.776	*	(0.804)	-1.811		(1.954)
**Structure and environment characteristics**										
Generic share (DDD)	1.641		(2.343)	Generic share (DDD)	6.631	**	(1.865)	16.893	***	(4.258)
Market concentration (HHI)	-0.082		(1.068)	Market concentration (HHI)	0.176		(1.864)	2.431		(1.348)
**Year after registration**										
Year 1	2.036	***	(0.219)	Year 1	3.446	***	(0.509)	2.200	***	(0.245)
Year 2	2.995	***	(0.252)	Year 2	4.639	***	(0.700)	3.091	***	(0.222)
Year 3	3.289	***	(0.268)	Year 3	5.145	***	(0.874)	3.502	***	(0.243)
Year 4	3.582	***	(0.296)	Year 4	5.543	***	(0.867)	3.594	***	(0.238)
Year 5	4.068		(0.266)	Year 5	6.322	***	(0.712)	3.788	***	(0.224)
R – squared	0.170			R – squared	0.518			0.005		
Corr (u_i, Xb)	-0.635			Corr (u_i, Xb)	-0.541			-0.755		
Rho	0.971			Rho	0.903			0.995		
Number of observations	142			Number of observations	65			77		

* *P* < 0.05; ** *P* < 0.01; *** *P* < 0.001

For ATC L category, we found that the price elasticity is similar to regression for all therapeutic groups. The price elasticity is between 0.8 and 1.6, which showed that price was elastic in the public sector but inelastic in the private sector. Generic use at ATC-4 was significantly associated with NCE utilization in the public sector but not in the private sector. Market concentration at ATC-4 was also significantly negative related to utilization in the public sector but was not significant in the private sector. From the sector specific regression, trends showed that the changes in the rate of diffusion was larger in the private sector while changes in the rate of diffusion was smaller in the public sector. The results demonstrated that public sector regression strongly influence the overall results for ATC L category.

Price elasticity for cardiovascular system (ATC C) was more elastic than results found for antineoplastic therapeutic groups (ATC L) between 1.7 and 1.8 which showed that price was more elastic in the private sector than the public sector. Generic share was significantly positive in cardiovascular medicines and this result was significantly more in the private sector which indicated that increasing generic medicines usage does not lead to less diffusion of NCEs. The result also showed that changes in the rate of diffusion was slightly higher in the public sector while changes in the rate of diffusion was smaller in the private sector.

In summary, the overall results revealed heterogeneity across therapeutic groups and both drug and market environment characteristics which affects the diffusion of NCEs were different across these therapeutic groups. By analysing different sectors separately, we also found differences in both drug and market environment characteristics that affects the diffusion of NCEs.

## Discussion

The analyses of the diffusion of innovative pharmaceutical products in this mixed market environment confirmed many earlier findings on the factors associated with diffusion. Regardless of sector and specific ATC category, price was a significant determinant of demand and speed of diffusion. A higher price is expected to lower the speed of diffusion [25]. To give some contexts, Ferrario [39] found the price per DDD for oncology medicines in Scotland, Belgium and Sweden had a regressive effect on utilization while Berndt et al. [16] observed that price elasticity was negative about − 1.1 for antihypertensives and highly significant. The analyses here further find sectoral differences in the magnitude of the price effect. The private sector, where the patient is exposed to the implications of prices (the vast proportion of private healthcare is paid out – of - pocket rather than through private insurance or through employers) is more sensitive to pricing. However, price discrimination in private sector that remains unchanged to demand, is unlikely to be feasible when medicines are sold to largely self-pay patients. The effect maybe larger as the prices used in the dataset was prices purchased by health facilities and not patient’s prices. Addressing price differential in the private sector is crucial as it can represent a barrier to access due to financial constraints. The price effect is linked to organizational factors on medicines procurement model. Private healthcare sector consists of group or individual facilities with fragmented procurement, hence less purchasing power to negotiate prices from the pharmaceutical suppliers. On the other hand, the higher demand of elasticity in the public sector was more likely due to payers implementing cost containment initiatives due to allocation of fixed budgets to meet increasing demand of patients, such as medicines procurement via national tenders to obtain economies of scale and price negotiations with pharmaceutical companies.

Evidence has shown that generic share is expected to negatively affect the diffusion of NCEs [15]. The generic medicines policy is used extensively in the public sector to support cost containment initiatives and push down prices over time. However, this effect was not seen in the private sector as the generic medicines policy is not mandatory in the private sector. In contrast, analysis in ATC C category showed generic share had a positive relationship with NCE uptake. This may be due to additional value in the NCE products which is more superior, for example characteristic of new molecules that is novel or combination products / new dosage forms that helps increase compliance thus provide meaningful benefit to patients.

Market concentration has a strong and significant association with diffusion of new medicines [34, 37]. However, this association was only observed significantly in the private sector all - class regression model and public sector in ATC L category. Market concentration of products depends on the coverage decision to be included in the prescriber / formulary list. Public health sector has a centralized medicines formulary committee that decides on coverage decisions within the system. A set of criteria such as cost effectiveness and budget implications of new medicines replacing existing alternatives are evaluated before inclusion in the prescriber / formulary list [40]. The overall effect was not significant which may indicate that the overall prescriber / formulary list is not highly concentrated and not restrictive, at least in the Malaysia public healthcare system. Private hospitals each has its own prescriber / formulary list that varies according to individual prescribers. In summary, market concentration provided mixed results depending on sector and ATC category.

Private healthcare market is market - based economy, where the process of supply, demand and pricing operate with minimal government involvement. Hence, the environment without prescribing restrictions coupled with private financing and fee – for - service has allowed physicians to freely prescribe newly marketed medicines. This was evident by the larger utilization of NCEs in the private sector. The diffusion of NCEs in the private sector was largely influenced by market competitiveness and lower price elasticity of demand at provider level, and may also be influenced by unobserved factors such as prescriber’s characteristics, marketing strategies or organisational related factors that are not captured in the analysis. Public sector, on the other hand, needs to ensure financial sustainability of the healthcare system and value for money in order to provide access to new and proven cost - effective medicines. This is due to the fact that Ministry of Health services are accessible for a nominal user fee, highly subsidised and does not cover the actual cost of services. Hence, higher price elasticity of demand at provider level and larger generic share utilization was shown to influence the diffusion of NCEs in the public sector. As observed in the findings, not all NCEs adopted in the private sector were adopted by the public sector. Selection of medicines should be targeted to medicines that brings additional value and limit the use of medicines with uncertain benefits or moderate therapeutic value to existing therapies. This would ensure resources are not drawn away from potentially more effective interventions.

The study found that the adoption of new medicines mostly occurred during the first year after registration for both sectors. These findings differ to some arguments that the uptake of new medicines in the public sector is limited [41]. Even though utilization was larger in the private sector, the diffusion speed was higher in the public sector over time. This may be due to coverage decisions at the national level spread through network of information across public health facilities. Coverage decisions where new medicines are cost effective shifts the volume from older medicines with lower therapeutic value to cost effective interventions. Comparatively, the speed of diffusion over time in the private sector may be limited due to price effect.

The sectoral difference in diffusion of NCEs showed that access to new medicines is not uniform and may not be equitable among patients in the country. This creates a scenario whereby patients that seeks initial treatment (e.g. early cancer treatment) in the private sector may be referred to public hospitals where the new medicines prescribed may not be available in the public sector. Hence, the decision may be either to adopt the new medicines subject to available resources or request patients to purchase outside of the public health system. Due to this circumstances, there is a need to reduce the differential access to new medicines between public and private sectors. The adoption of new medicines in individual markets should be balanced between a higher investment in pharmaceutical budget and the evidence of cost - effectiveness. One way is to create policy environment that adopt policies holistically across sectors such as health technology assessment and generic medicines policy to increase the efficient use of resources. A future national reimbursement system from pooled financing that does effective strategic purchasing for both sectors may help to smoothen out the differential delays and govern access to cost effective treatments, furthermore, creating better purchasing power to negotiate prices with pharmaceutical manufacturers for the whole country. Our findings correlate with the current public - private health service delivery and health financing sytem. Therefore, a systematic shift between both sectors in these areas to be more aligned in tackling disease burden is necessary and may help to improve the equitable access to new medicines. This can be done by strengthening the cooperation around a shared vision of sustainable healthcare [42].

## Conclusions

Our study provides empirical evidence towards perspectives that were intuitively known from having a dual healthcare market and the findings may possibly extend to similar health care system in other countries. Several limitations were found in our study. Firstly, our study did not include other important factors that may further explain the difference in diffusion of NCEs between sectors, such as, marketing strategies and prescriber behaviours. We hope additional analysis at patient level data will help support our findings. It was impossible to include health outcome measurement of new medicines that may provide explanation on the uptake of these medicines and we propose that this variable be included when such data is available. Our study includes hospitals under Ministry of Health and university hospitals that may have different characteristics, hence analysis on different diffusion trends is also recommended. Lastly, we have performed the analysis to the best of our ability, however other methods that may be available to measure diffusion of medicines may be conducted in future work. More evidence is still required to balance the dual healthcare market dynamics and carve effective health policy mix needed to address the current disease burden of the country. Furthermore, understanding the impact of access to new medicines in a mixed market is necessary to ensure efficient use of healthcare resources for cost effective medicines.

## Supplementary Information

**Additional file 1.**

## Data Availability

The National Pharmaceutical Audit data that supports the findings of this study is available from IQVIA (formerly known as QuintilesIMS) Malaysia. Restrictions apply to the availability of this data, which was used under license for the current study, and is not publicly available. Data is however available from the authors upon reasonable request and with permission from IQVIA Malaysia. The Pharmaceutical Product Registration Database is available from the National Pharmaceutical Regulatory Agency, Ministry of Health Malaysia upon reasonable request.

## Abbreviations

ATC: Anatomical Therapeutic Chemical
DDD: Defined Daily Dose
FE: Fixed effect
GDP: Gross domestic product
HHI: Herfindahl – Hirschman Index
MYR: Malaysian Ringgit
NCE: New Chemical Entity
NPRA: National Pharmaceutical Regulatory Agency
OLS: Ordinary Least Square
RE: Random effect

## Footnotes

2 The DDD is calculated as the average maintenance dose per day for a drug used for its main indication in adults.

3 MYR = Malaysian Ringgit; equivalent to approximately USD 0.23.

4 ATC4 is one level below the therapeutic class and is specific to a therapeutic class functioning in a similar manner.
