# Supplementary material for: Differential diffusion of pharmaceutical innovations in a mixed market middle - income economy
Source: BMC Health Serv Res. 2021 Oct 19;21:1119. doi: 10.1186/s12913-021-06786-6 (PMC8524869; doi:10.1186/s12913-021-06786-6)
Supplement: Supplementary file 1 — Additional file 1. [file 12913_2021_6786_MOESM1_ESM.docx]

Annex

Table 1: Statistical test results for pooled OLS, random effect and fixed effect panel regression models.

|  | Model 1 | | | ATC C | | | ATC L | | |
| --- | --- | --- | --- | --- | --- | --- | --- | --- | --- |
|  | Pooled OLS | Random Effect | Fixed Effect | Pooled OLS | Random Effect | Fixed Effect | Pooled OLS | Random Effect | Fixed Effect |
| Breusch-Pagan LM test | 810.69  (0.0000)*** | |  | 114.72  (0.0000)*** | |  | 83.87  (0.0000)*** | |  |
| Hausman test |  | 68.42  (0.0000)*** | |  | 194.14  (0.0000)*** | |  | 164.67  (0.0000)*** | |
| Chow Test | 101.33 (0.0000)*** | | | 212.95 (0.0000)*** | | | 9.01 (0.0030)** | | |
| Multicollinearity (VIF) |  |  | 2.34 |  |  | 2.25 |  |  | 2.41 |
| Heteroskedasticity (Modified Wald Statistic |  |  | 1.0e+34  (0.0000)*** |  |  | 1.6e+31  (0.0000)*** |  |  | 1.1e+33  (0.0000)*** |
| Serial correlation |  |  | 126.319  (0.0000)*** |  |  | 39.713  (0.0000)*** |  |  | 20.246  (0.0001)*** |

Notes: Figures in the parentheses are p-value.
